# HIV Acquisition Is Associated with Increased Antimicrobial Peptides and Reduced HIV Neutralizing IgA in the Foreskin Prepuce of Uncircumcised Men

**DOI:** 10.1371/journal.ppat.1004416

**Published:** 2014-10-02

**Authors:** Taha Hirbod, Xiangrong Kong, Godgrey Kigozi, Anthony Ndyanabo, David Serwadda, Jessica L. Prodger, Aaron A. Tobian, Fred Nalugoda, Maria J. Wawer, Kamnoosh Shahabi, Olga L. Rojas, Jennifer L. Gommerman, Kristina Broliden, Rupert Kaul, Ronald H. Gray

**Affiliations:** 1 Infectious Diseases Unit, Department of Medicine Solna, Karolinska University Hospital, Karolinska Institutet, Stockholm, Sweden; 2 Department of Epidemiology, Johns Hopkins University, Bloomberg School of Public Health, Baltimore, Maryland, United States of America; 3 Rakai Health Sciences Program, Kalisizo, Uganda; 4 School of Public Health, College of Medicine, Makerere University, Kampala, Uganda; 5 Departments of Medicine and Immunology, University of Toronto, Toronto, Canada; Emory University, United States of America

## Abstract

**Background:**

The foreskin is the site of most HIV acquisition in uncircumcised heterosexual men. Although HIV-exposed, seronegative (HESN) uncircumcised men demonstrate HIV-neutralizing IgA and increased antimicrobial peptides (AMPs) in the foreskin prepuce, no prospective studies have examined the mucosal immune correlates of HIV acquisition.

**Methods:**

To assess the association of foreskin immune parameters with HIV acquisition, antimicrobial peptides and IgA with the capacity to neutralize a primary clade C HIV strain were quantified by blinded investigators, using sub-preputial swabs collected longitudinally during a randomized trial of male circumcision for HIV prevention in Rakai, Uganda.

**Results:**

Participants were 99 men who acquired HIV (cases) and 109 randomly selected controls who remained HIV seronegative. At enrollment, 44.4% of cases vs. 69.7% of controls demonstrated IgA neutralization (adjusted OR = 0.31; 95% CI, 0.16–0.61). IgA neutralization was detected in 38.7% of cases and 70.7% of controls at the last seronegative case visit prior to HIV acquisition and the comparable control visit (adjusted OR 0.21; 95% CI, 0.11–0.39). Levels of the α-defensins and secretory leukocyte protease inhibitor (SLPI) were over ten-fold higher in the foreskin prepuce of cases who acquired HIV, both at enrollment (mean 4.43 vs. 3.03 and 5.98 vs. 4.61 log_n_ pg/mL, P = 0.005 and 0.009, respectively), and at the last seronegative visit (mean 4.81 vs. 3.15 and 6.46 vs. 5.20 log_n_ pg/mL, P = 0.0002 and 0.013).

**Conclusions:**

This prospective, blinded analysis is the first to assess the immune correlates of HIV acquisition in the foreskin. HIV-neutralizing IgA, previously associated with the HESN phenotype, was a biomarker of HIV protection, but other HESN associations correlated with increased HIV acquisition. This emphasizes the importance of prospective epidemiological studies or *in vitro* tissue studies to define the impact of mucosal parameters on HIV risk.

## Author Summary

Randomized trials of male circumcision (MC) for HIV prevention have shown that the foreskin is a major site of HIV acquisition among heterosexual men, but a number of barriers to MC programs mean that many HIV-susceptible men remain uncircumcised. The immune correlates of HIV acquisition in the foreskin are poorly described, and may inform prevention strategies for uncircumcised men. Using swabs collected prospectively during a previous randomized trial of MC for HIV prevention in Uganda, blinded investigators assessed levels of HIV-neutralizing IgA and soluble antimicrobial peptides in the foreskin prepuce of cases who acquired HIV (n = 99) and controls men who did not (n = 109). We show that IgA with the capacity to neutralize HIV was associated with protection against HIV, while levels of α-defensins and secretory leukocyte protease inhibitor (SLPI) were associated with increased acquisition. This is the first study to assess the immune correlates of HIV acquisition in the foreskin. Advantages included its relatively large sample size, rigorous blinding of immune assays, and ability to control for relevant potential confounders.

## Introduction

During insertive sex, the tissue sites where a man may acquire HIV-1 (HIV) include the penile shaft, foreskin and urethral meatus [1]. Three independent randomized clinical trials demonstrated that male circumcision reduces the risk of HIV acquisition by 50–60% [2]–[4], and considerable additional clinical and biologic evidence points to the foreskin as being the major site for initial HIV infection in uncircumcised men [5]–[13]. While other penile tissues play a role, both before and particularly after circumcision, their overall importance in HIV acquisition is less clear [1]. Despite the scale of the HIV pandemic, the probability of female-to-male HIV transmission is just under 1/250 per coital act in low-income countries [14], although there is heterogeneity in this risk depending on factors such as genital ulcer disease (GUD) in either partner, and the HIV viral load (VL) in the blood and genital tract of a man's sexual partner [15]. This relatively low per-contact probability of HIV transmission means that the great majority of penile HIV exposures do not lead to productive infection, and implies that innate or adaptive host immune defences may play a role in natural protection against HIV [16].

Antimicrobial peptides (AMPs) are soluble innate immune factors present at high levels in human mucosal secretions, and may have anti-HIV activity *in vitro* [17], [18]. Specific antimicrobial peptides that possess HIV inhibitory activity *in vitro* and are present at physiologically relevant levels in genital secretions include the human neutrophil peptides 1–3 (HNP1–3) that are members of the human α-defensins family [19], [20], the cathelicidin LL-37 [21], and the serine protease inhibitor secretory leukocyte protease inhibitor (SLPI) [22]. While these immune factors have been most studied in the female genital tract, α-defensins (HNP1–3 and HD5), β-defensins (HBD2), SLPI and lactoferrin are also present in male urethral secretions [23], [24]. Less is known about the presence and/or levels of AMPs in the foreskin prepuce. While HIV-exposed seronegative (HESN) Ugandan men had increased α-defensin levels at this site, suggesting a possible role in immune protection against HIV [25], an increased level in the female genital tract was associated with genital co-infections and increased HIV acquisition [26].

In addition to AMPs, there is abundant secretory immunologlobulin A (IgA) in male urogenital mucosal secretions [24]. Furthermore, mucosal IgA from the genital and oral secretions of some HESN men who have sex with men [27], HIV-discordant couples [28] and female commercial sex workers [29] has been shown to have the capacity to neutralize HIV *ex vivo*, and the frequency of HIV-neutralizing IgA was increased in the foreskin prepuce of HESN Ugandan men [25]. Importantly, the specificity of this IgA remains unknown [30] and detection of HIV-specific IgA by ELISA is relatively rare [31], [32]. While not all investigators have found genital HIV-neutralizing IgA in HESN populations, the strongest evidence for their role in HIV protection comes from a blinded, prospective clinical study in high-risk Kenyan women [33].

Since innate AMPs and neutralizing IgA were both increased in foreskin prepuce of HESN men in Uganda [25], we hypothesized that these mucosal immune factors would protect against female-to-male heterosexual HIV transmission during insertive vaginal sex. To test this hypothesis, these immune parameters were assayed by rigorously blinded researchers in a nested case-control study format, using preputial secretions that had been collected prospectively from uncircumcised men during a randomized clinical trial of male circumcision (MC) for HIV prevention in Rakai, Uganda [4].

## Results

### Clinical and behavioral characteristics of study participants

Participants were HIV seronegative, uncircumcised men participating in a previously-described clinical trial of male circumcision for HIV prevention [4]. Cases (N = 99) subsequently acquired HIV, while controls (N = 109) remained HIV seronegative throughout the trial and early post-trial follow up. The clinical and demographic characteristics of cases and controls were broadly comparable at enrollment (Table 1). However, in keeping with an increased HIV risk, cases were more likely than controls to be never or previously married, reported a higher number of sexual partners and non-marital partners in the past year, and were more likely to report consumption of alcohol prior to intercourse. Cases were also more likely to be engaged in non-agricultural employment. Therefore, all subsequent multivariable regression models were specifically adjusted for these covariates, as well as for subsequent circumcision (see Methods).

**Table 1:** Demographic characteristics of cases and controls at enrollment.

Characteristics and behaviors	Cases N = 99		Controls N = 109		P-value
	N	(%)	N	(%)	
**Age**					
15–24	44	(44.4)	55	(50.5)	0.55
25–29	29	(29.3)	25	(22.9)	
30–49	26	(26.3)	29	(26.6)	
**Marital status**					
Never married	52	(52.5)	51	(46.8)	0.05
Currently married	35	(36.4)	53	(48.6)	
Previously married	12	(12.1)	5	(4.6)	
**Occupation**					
Agriculture	45	(45.5)	60	(55.1)	0.005
Student	9	(9.1)	21	(19.3)	
Other	45	(45.5)	28	(26.7)	
**Number of sex partners past year**					
None	5	(5.1)	22	(20.2)	<0.001
1	40	(40.4)	52	(47.7)	
2+	54	(54.5)	35	(32.1)	
**Number of Non-marital sex partners past year**					
None	30	(30.3)	57	(52.3)	0.003
1	39	(39.4)	35	(32.1)	
2+	30	(30.3)	17	(15.6)	
**Intercourse past week**	51	(51.5)	49	(45.0)	0.34
**Condom use in the past year in sexually active**					
None	36	(38.3)	38	(43.7)	0.06
Inconsistent	46	(48.9)	29	(33.3)	
Consistent	12	(12.8)	20	(23.0)	
**Alcohol before sex**	57	(60.6)	40	(46.0)	0.05
**Symptoms of STIs**					
GUD	3	(3.0)	2	(1.8)	0.67
Genital discharge	2	(2.0)	0	(0)	0.23
Dysuria	2	(2.0)	2	(1.8)	1.00

### IgA with HIV-neutralizing capacity was associated with HIV protection

At enrollment, HIV was neutralized by IgA purified from the foreskin preputial swabs of 44.4% of cases and 69.7% of controls (adjusted OR: 0.31, 95% CI 0.16–0.61), meaning that the lack of IgA neutralizing capacity was associated with a 3.23 increased risk of seroconversion (adjusted 95% CI 1.64–6.25; Table 2). Using the seronegative sample that had been collected most proximal to the time of HIV infection (for cases) and the control samples from the same visits, the adjusted odds ratio of HIV acquisition in the absence of IgA neutralization was further increased to 4.76 (adjusted 95% CI 2.56–9.09). Secretory IgA was then quantified in samples with sufficient remaining material (n = 77), giving a median sIgA concentration of 46 ng/ml (range, 30–580 ng/ml) among the positive samples. There were no significant differences between the study groups, and the quantity of sIgA did not correlate with presence of HIV neutralizing capacity (P>0.2). There was insufficient material to also measure total IgA for calculation of an sIgA/IgA ratio.

**Table 2:** Sub-preputial HIV neutralizing IgA and HIV acquisition.

Sample timing	HIV neutralization^a^ (n/N; %)		Univariate OR^b^ (95% CI)	Adjusted OR^b^^,^ c (95% CI)
**Enrollment**				
Cases	44/99	(44.4)	0.35 (0.20,0.61)	0.31 (0.16, 0.61)
Controls	76/109	(69.7)	1.00	1.00
**Last case seronegative sample**				
Cases	36/93	(38.7)	0.26 (0.15–0.45)	0.21 (0.11–0.39)
Controls	116/166	(70.7)	1.00	1.00

a HIV neutralization based on a cut off of ≥67%.

b Estimates obtained using GEE accounting for the repeated measurements.

c Adjusted for occupation, marital status and time-dependent number of sex partners, condom use and alcohol use with sex.

Our standard neutralization assay defines HIV-neutralizing capacity based on a reduction of HIV replication in PBMCs of ≥67%. A sensitivity analysis using the more conservative neutralization cutoff of ≥90% found an even stronger association between HIV acquisition and the lack of HIV-neutralizing IgA. Specifically, 60.6% of cases (60/99) and 31.2% of controls (34/109) lacked HIV neutralization by IgA at enrollment, with an adjusted odds of HIV acquisition of 3.85 (95% CI 2.00–7.69). Similarly, using the ≥90% neutralization for the foreskin swab collected most proximal to the HIV acquisition visit, absence of neutralization was detected in 63.4% of cases (59/93) and 34.1% of controls (56/164; adjusted OR 4.17, 95% CI 2.27–7.69).

There were 48 cases whose last seronegative visits were at 6 or 12 months, and 11 of these participants (22.9%) had HIV neutralization by IgA at enrollment, but not at their last seronegative visits. Among the corresponding controls (N = 55), 10 (18.2%) had HIV neutralization by IgA at enrollment but not at their follow-up visits. Thus, a decline in IgA neutralization capacity was not significantly associated with HIV seroconversion (P = 0.55).

### Foreskin AMP levels and HIV acquisition

**Figure 1:**
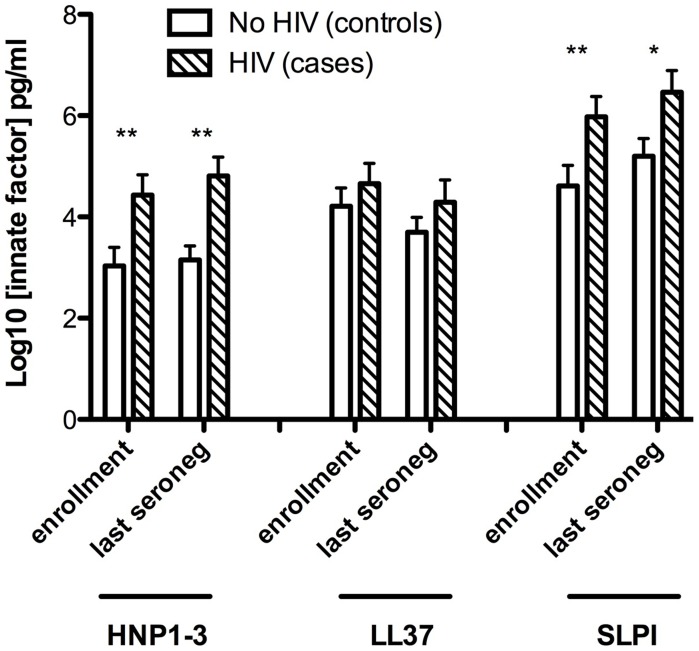
Sub-preputial antimicrobial peptide (AMP) levels and HIV acquisition. Foreskin prepuce levels of the soluble antimicrobial peptides HNP1–3 (X axis, left), LL37 (X axis, center) and SLPI (X axis, right). Levels are shown on the Y axis as log10 transformed data, and were assayed for cases (n = 99, hatched bars) and controls (n = 109, white bars) at both the enrollment visit (left pair for each AMP) and the last visit prior to seroconversion (right pair for each AMP).

Levels of alpha-defensins (HNP1–3), beta-defensins (HBD2), lactoferrin, LL37 and SLPI were assessed in preputial swab secretions at enrollment and at the last case seronegative visit (Figure 1). Levels of β-defensins and lactoferrin in sub-preputial swabs fell below the lower limit of assay detection (7.8 and 400 pg/mL, respectively) in all participants, and so were not assessed.

Cases who subsequently acquired HIV had significantly higher levels of HNP1–3 than controls, both at enrollment (mean 4.43 vs. 3.03 log_n_ pg/mL, respectively, P = 0.005) and at the last case seronegative visit (mean 4.81 vs. 3.15 log_n_ pg/mL, respectively, P = 0.0002). Furthermore, SLPI levels were also significantly higher in cases than controls at enrollment (mean 5.98 vs. 4.61 log_n_ pg/mL, P = 0.009) and the last case seronegative visit (mean 6.46 vs. 5.20 log_n_ pg/mL, P = 0.013). No statistically significant differences between cases and controls were detected in levels of LL37.

Table 3 shows the frequency of detection and the odds ratios of detectable AMPs associated with HIV acquisition. HNP1–3 was more frequently detected among cases than controls, both at enrollment (adjusted OR 2.89, 95% CI 1.49–5.60) and at the last case seronegative sample (2.39, 95% CI 1.40–4.07). There was no association between the detection of LL37 at enrollment and subsequent HIV acquisition, but detection of SLPI at the last case seronegative visit was statistically significantly associated with HIV acquisition (adjusted OR 2.05, 95%CI 1.14–3.71).

**Table 3:** Frequency of detection of antimicrobial peptides and HIV acquisition.

Antimicrobial peptide, study visit	Detectable N (%)	Undetectable N (%)	Univariate OR^a^ (95% CI)	Adjusted OR^a,b^ (95% CI)
**HNP1–3**				
**Enrollment**				
Cases:	59 (59.6)	40 (40.4)	2.0 (1.15–3.49)	2.89 (1.49–5.60)
Controls:	45 (42.5)	61 (57.6)	1.00	1.00
**Last case seronegative sample**				
Cases:	64 (69.6)	28 (30.4)	2.46 (1.43–4.24)	2.39 (1.40–4.07)
Controls:	77 (48.1)	83 (51.2)	1.00	1.00
**LL37**				
**Enrollment**				
Cases:	60 (60.6)	39 (39.4)	1.07 (0.61–1.87)	1.00 (0.53–1.89)
Controls:	62 (59.1)	43 (41.0)	1.00	1.00
**Last case seronegative sample**				
Cases:	48 (52.2)	44 (47.8)	1.00 (0.59–1.65)	1.01 (0.60–1.71)
Controls:	83 (52.5)	75 (47.5)	1.00	1.00
**SLPI**				
**Enrollment**				
Cases:	74 (74.8)	25 (25.3)	2.10 (1.16–3.81)	1.85 (0.94–3.64)
Controls:	62 (59.1)	43 (41.0)	1.00	1.00
**Last case seronegative sample**				
Cases:	72 (78.3)	20 (21.7)	2.10 (1.17–3.80)	2.05 (1.14–3.71)
Controls:	101 (63.1)	59 (36.9)	1.00	1.00

### Foreskin tissue contains IgA+CD138+ cells

**Figure 2:**
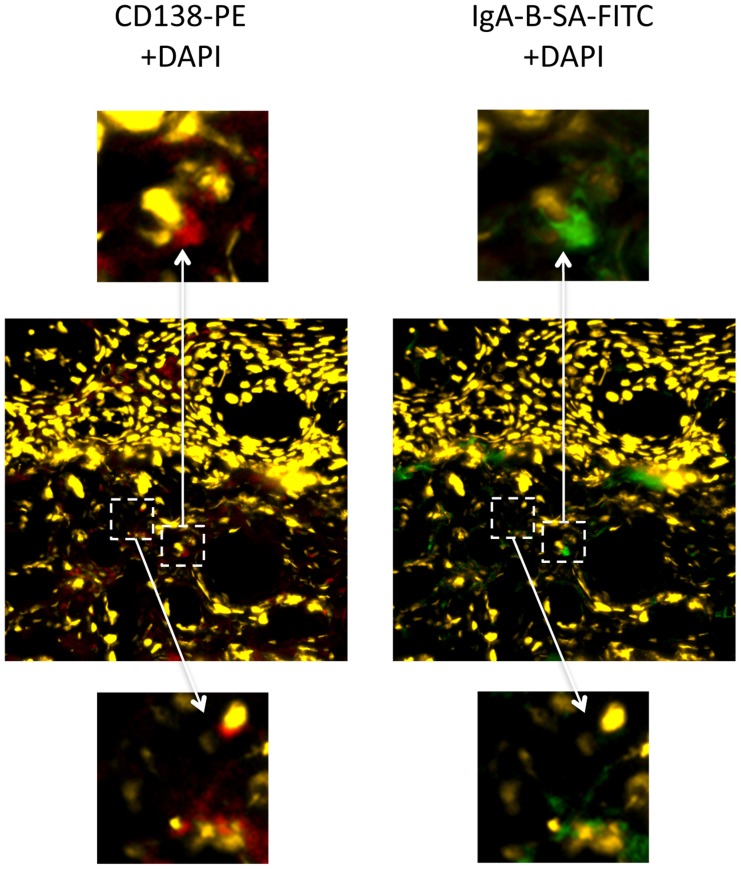
IgA+CD138+ cells in the foreskin of an HIV-uninfected Ugandan man. Foreskin tissues collected from an HIV-uninfected, low risk Ugandan man were cryopreserved within 10 minutes of surgery and shipped to Toronto, Canada for immunofluorescence microscopy. IgA+CD138+ cells were present in the foreskin dermis. Central panels demonstrate staining with CD138 PE (red) and DAPI for nuclei (yellow) at 20× magnification (left) and staining with IgA biotin-SA FITC (green) and DAPI (yellow) at 20× magnification (right). Dashed white boxes and arrows highlight two sites of interest at 80× magnification: upper panels highlight a cell staining positive for both CD138 (red; left) and IgA (green; right), while lower panels highlight a cell positive only for CD138.

Cryopreserved tissues from trial participants were not available for study. Therefore, in order to investigate a possible local source of IgA production in the foreskin, we performed immunofluorescence studies using foreskin tissues that had been obtained from two HIV-uninfected Ugandan man after elective MC and cryopreserved within ten minutes. Both men were putatively HIV-unexposed; they were enrolled in the Rakai Community Cohort Study, and reported that they had been in a monogamous relationship for at least one year with a female partner confirmed to be HIV-uninfected. In one participant, IgA expression was clearly demonstrated in occasional cells that co-expressed CD138 in the foreskin dermis, with a density of approximately 1–2 IgA+CD138+ cells/µm^2^ (representative example, Figure 2). Distribution of these cells was heterogeneous within the foreskin tissue examined, and IgA+CD138+ cells were not seen in the foreskin of the second participant.

## Discussion

The foreskin is the predominant site of HIV acquisition in uncircumcised heterosexual men, and a possible protective role for sub-preputial HIV-neutralizing IgA and α-defensins against HIV acquisition was suggested by recent studies showing that both parameters were elevated in the foreskin prepuce of HESN men [7]. However, cross-sectional studies of HESN individuals are subject to important potential confounders [25], and to our knowledge no prospective studies have previously examined the immune correlates of HIV protection in the foreskin using HIV acquisition as the primary endpoint. In this nested case-control study, with all immune assays performed in a rigorously blinded fashion, we found that HIV acquisition was associated with a lack of foreskin IgA with HIV-neutralizing capacity in the foreskin sub-prepuce, but with increased levels of α-defensins and SLPI at this site. Interestingly, these findings closely mirror those of a previous blinded, nested case-control study of HIV acquisition in high-risk women from Kenya [33]. In the latter study, the odds ratio of HIV infection among participants with IgA neutralization in cervico-vaginal secretions was 0.31, almost identical to the association with foreskin IgA neutralization in this study. Furthermore, a robust association between HIV acquisition and increased mucosal α-defensin levels was observed in both male and female studies.

The current study has several strengths. Perhaps most important in terms of the robustness of our conclusions was the prospective nature of the study and the rigorous blinding of laboratory investigators to participant group. In addition, the large number of participants increased our statistical power, and was possible due to nesting of the study within a large randomized clinical trial that had used HIV acquisition as an endpoint. The latter also meant that extensive epidemiological and clinical data were available, and allowed us to carefully adjust for potential confounders in multivariate analyses. Although data regarding HIV sexual exposure were not formally collected during the trial, it is unlikely that the increased HIV-specific IgA in controls was due to an over-representation of HESN men in this group, since (as might be expected) self-reported sexual risk was actually higher among cases who acquired HIV, and among the subset of controls with data available through the Rakai Community Cohort Study, only 2% self-reported an HIV-infected female partner [Gray R, personal communication]. Penile wetness has been associated with increased HIV susceptibility [34], [35]. While wetness was not assessed in our study, overall sIgA concentrations did not vary between cases and control groups, suggesting that there was no major difference in dilution of mucosal antibodies.

The nature and source of the neutralizing IgA that we found in the sub-prepuce are not yet fully elucidated. IgA1 and IgA2 isoforms are often present as dimeric or polymeric sIgA in mucosal secretions, having acquired J chain during synthesis and secretory component during epithelial transport. Whereas cervical mucus contains mostly polymeric IgA, vaginal fluid contains both monomeric and dimeric IgA [36]. In the present report a proportion of the IgA1 antibodies in the sub-preputial secretions were of the sIgA type. Ig-producing plasma cells and polymeric Ig receptor expression are present in the penile urethra, along with abundant IgA and secretory component molecules in the mucus layer overlying the urethral epithelium [37], and the urethral meatus is in close proximity to the foreskin in the unerect penis. Our immunofluorescence studies demonstrated numerous cells that co-expressed IgA and CD138, a plasma cell marker, and IgA in foreskin tissue from an HIV-uninfected, low-risk Ugandan man. However, their distribution was heterogenous and no IgA+CD138+ cells were seen in foreskin tissues from a second participant. Inter-individual and intra-individual heterogeneity in the distribution of IgA+CD138+ cells, as well as in the presence of HIV-neutralizing IgA in the foreskin, might relate to a lack of plasma cell (PC) survival factors such as APRIL and BAFF, preventing protracted PC residence at this site. Future studies will be needed to define the frequency, phenotype and distribution of IgA+ cells in the foreskin, but our results do suggest that HIV-neutralizing IgA antibodies in the sub-prepuce may be derived from either the foreskin tissue itself, from the urethra, or via serous transudation from foreskin vasculature.

Both the current study and our previous work in high-risk women suggest that the neutralizing IgA detected may not be induced by prior antigen exposure, since it may be observed in lower risk participants with no known HIV exposure, and the rates of gp120 ELISA reactivity are very low [30]. The specificity of the HIV neutralizing sub-preputial IgA antibodies remains unknown, and this constitutes an important area for future study. HIV-inhibitory IgA antibodies in the genital secretions of HIV exposed seronegative women target various regions of HIV gp41, as well as CCR5, and the clade C neutralization we observed suggests that conserved viral and cellular structures might also be involved [33], [38]–[41]. Indeed, this may explain discrepancies regarding the lack of detectable mucosal IgA in binding assays (eg: ELISA) as opposed to detection of HIV neutralizing IgA in HESN individuals [42].

Mucosal antiviral activity was thus assessed by HIV neutralization in a PBMC-based assay, which may underestimate antiviral capacity. A number of other potential mechanisms of viral inhibition, mediated by both antibodies and AMPs [18], merit exploration in future studies. These include immune molecules directed against both viral and host targets involved in the initial steps of HIV entry in the foreskin, such as Langerhans cells [9]. However, the IgA-mediated neutralizing activity that was assayed should be very relevant, since this allows detection of antibodies directed against both viral and cellular targets. The neutralization data also implicate a cross-clade neutralizing response, since HIV clades A and D predominate in the region [43], [44], while HIV subtype C was used in the neutralization assay. Since different primary isolates may vary considerably in their neutralization sensitivity, definitive conclusions regarding clade and/or epitope specificity of genital HIV-neutralizing IgA require data from a broader panel of primary HIV isolates. The low amounts of IgA present in the sub-preputial secretions did not permit a panel approach in the current study, so we opted to use the clade C isolate as a possible marker of broad HIV neutralizing capacity.

In addition to IgA, numerous mucosal AMPs demonstrate anti-HIV activity *in vitro* [17], [18]. Such mucosal AMPs with anti-HIV activity in vitro could theoretically have either beneficial or detrimental effects in vivo. Effects of AMPs include the blockade of virus entry and subsequent infection [20], [45], [46], and/or the augmentation of adaptive immune responses through the recruitment of immune cells [47]. However, since some of the immune cells that are recruited are HIV-susceptible, particularly CD4+ T cells, this could indirectly increase the risk of HIV acquisition. Whether the direct HIV inhibitory effect of these factors overrides the potentially negative effects on HIV susceptible target cell recruitment requires further study. In this case–control study we found that higher levels of the α-defensins HNP1–3 and SLPI in the foreskin prepuce were associated with increased HIV acquisition. These findings do not prove that these AMPs directly enhanced HIV acquisition, and we did not formally assess the HIV neutralization capacity of these AMPs in the current work. It is possible that unmeasured behaviors associated with HIV risk are also associated with increases in genital AMPs, an important confounder to consider in studies of HESN individuals [48]. Nevertheless, the possibility that these AMPs may cause increased HIV susceptibility through local enhancement of HIV replication or through recruitment of HIV susceptible target cells [49] must be carefully considered, given the potential application of AMP analogues as microbicide candidates. Analogous to foreskin IgA, there are several potential sources for these AMPs, which may either represent local production by neutrophils and epithelial cells in the urethra and foreskin, or serous transudation within the highly vascularized foreskin.

A limitation of the present study is that only IgA1 was assessed for antiviral activity, since IgA2 does not bind to jacalin used for IgA purification. Prior studies have shown the importance of the IgA2 version of the monoclonal antibody 2F5 for inhibition in vitro of HIV transmission across mucosal surfaces [50]. In a related study, both the IgA1 and IgA2 version of the monoclonal antibody HGN194 neutralized the virus in an experimental non-human primate model [51]. Together these studies suggest that both IgA1 and IgA2 could have complementary and synergistic antiviral activities in mucosal secretions. While the jacalin-purification method for IgA has been previously validated [52], the possibility that additional jacalin-binding contaminants could contribute to the HIV neutralizing activity cannot be completely excluded. To minimize this possibility, including any contribution of the galactose eluant to antiviral activity, jacalin-purified samples from low-risk HIV seronegative controls were included in each assay.

In summary this prospective, blinded case-control study found that HIV acquisition in uncircumcised Ugandan men was associated with a reduced frequency of HIV-neutralizing IgA in the foreskin sub-prepuce, and with higher levels of the antimicrobial peptides HNP1–3 and SLPI. While important questions remain regarding the source and nature of the foreskin IgA, and the causal nature of the association between AMPs and HIV acquisition, these results have important implications for the HIV vaccine and microbicide fields. The fact that the results so closely mirror those of a blinded, prospective study in high-risk women [33], and that the strength of the association was increased proximal to the time of HIV acquisition, suggests that the effects are real and important.

## Materials and Methods

### Study population and infection diagnostics

A randomized trial of male circumcision to prevent HIV acquisition was performed from 2003–2006, among 4,996 initially uncircumcised HIV-seronegative men aged 15–49 from Rakai, Uganda [4]. Participants were randomized to either receive immediate circumcision or circumcision delayed for 24 months, and were followed up at 6, 12 and 24 months to detect incident HIV infection and to monitor sexual risk behaviors. At enrollment, all consenting men provided interview information on sociodemographic, behavioral and health characteristics and underwent a physical examination. Men with evidence of genital infections (urethral discharge, genital ulceration or dysuria), were treated and reassessed to ensure resolution of infection [4]. Blood samples were obtained for HIV, syphilis and herpes simplex type 2 (HSV-2) serology. The rapid plasma reagin test (RPR test, Becton Dickinson, Groot-Bijgaarden, Belgium) was performed to screen for syphilis, and positive samples confirmed by Treponema pallidum particle agglutination assay (Serodia TP-PA kit, Fujirebio, Malvern, USA). Testing for HSV-2 was performed using an HSV-2 IgG enzyme immunoassay (Kalon Biological, Guildford, UK). HIV status was determined by two enzyme immunoassays (EIAs); Vironostika HIV-1/2 Plus O (Organon Teknika, Charlotte, NC, USA) and Murex HIV-1.2.0 (Murex Biotech Limited, Dartford, UK), run in series. All samples concordant or discordant on the two EIAs, were subjected to Western blot (WB) confirmation (HIV-1 Western Blot; Bio-Merieux-Vitek) or by PCR (Amplicor HIV-1 Monitor Version 1.5, Branchberg, NJ, USA).

### Sample collection and IgA purification

Genital swab samples were obtained at enrollment and each follow up visit, using Dacron swabs moistened in sterile saline which were rotated twice around the full circumference of the penis at the coronal sulcus and glans, and were stored in AMPLICOR STD Specimen Transport Kit medium (Roche Diagnostic Systems, Branchburg, NJ, USA) at 4°C for less than 4 hours, until aliquoting and storage at −80°C.

IgA was purified from thawed preputial swab samples with jacalin/agarose as previously described [29] with minor modifications. Four hundred microliters of sub-preputial swab sample was added to 200 µL jacalin/agarose beads and mixed for 2 h at 48°C followed by centrifugation and collection of the IgA-depleted fraction. Jacalin/agarose beads were washed with PBS pH 7.4, and bound IgA was eluted overnight at room temperature in 0.5 mL 0.8 mol/L D-galactose pH 7.5. Purified IgA was collected and all fractions were stored at −80°C. At the time of standardizing the mucosal IgA assays the purity of the IgA fractions was examined, and no high-mass molecular weight proteins or residual IgG were detected by SDS-PAGE separation or Coomassie blue staining [Broliden K, personal communication]. An enzyme immunoassay was used for detection of sIgA with a detection limit of 20 ng/ml (sIgA ELISA Kit, Immundiagnostik AG, Germany).

### HIV neutralization assay

The neutralizing capacity of purified IgA was measured against a clade C HIV-1 R5 primary isolate (ZA97009) received from the NIH AIDS Reagent Program (https://www.aidsreagent.org). All neutralization assays were performed by investigators blinded to the HIV outcome. The primary isolate was only maintained in PBMC culture and were not passaged on cell lines at any time. A volume of 75 µL of purified IgA (diluted 1∶2) was incubated with 75 µL of the HIV-1 isolate (10–50TCID_50_) at 37°C for 1 h in 96 well plates and then cultured with 10^5^ PHA stimulated PBMC per well overnight. The plates were centrifuged and unbound virus was washed away. After 3 days 100 µL of the medium was discarded and replaced by fresh medium. The PBMCs were cultured for another 3 days. Supernatants of 100 µL were collected and analyzed for p24 antigen, using Murex HIV-1 antigen ELISA-kit (Abbott Diagnostics, Abbott Park, IL). Neutralization capacity was measured as inhibition of p24 antigen production and was considered positive when a two-third (67%) reduction was achieved compared with the mean results obtained from heat-inactivated plasma (diluted 1∶20) from four HIV uninfected controls. The selection of this neutralization cutoff was based on several prior studies [29], [53], [54]; to ensure that results were robust, a sensitivity analysis using a neutralization capacity cut off of ≥90% was also performed. Internal negative control samples were purified in the same way as the study samples, and included plasma and mucosal secretions from HIV seronegative low-risk Swedish controls; these were included to ensure that neutralizing activity was not mediated by galactose (from the IgA elution step) or any other non-IgA1 contaminant.

### Quantification of HNP1–3, LL-37, SLPI, HBD2 and Lactoferrin

Commercial enzyme-linked EIA kits were used to quantify the innate immune molecules of interest in the IgA-depleted fraction of the samples, according to the manufacturers protocols (additional dilution in brackets): HNP1–3 (1∶10), LL-37 (1∶5) (both Hycult biotechnology, Uden, the Netherlands), SLPI (1∶5) (RD Systems Europe Ltd, Abingdon Oxon, UK), HBD2 (1∶1) (Phoenix Pharmaceuticals, INC., Burlingame, USA) and Lactoferrin (1∶1) (Nordic biosite, Stockholm, Sweden). Again, all research personnel were rigorously blinded to participant group and clinical outcome.

### Immunofluorescence microscopy using foreskin tissues

Foreskin tissues from two consenting, low-risk Ugandan men were frozen in Optimal Cutting Temperature (OCT) within 10 minutes of MC surgery and shipped to Toronto for analysis. Staining for IgA and the plasma cell marker CD138 was performed: foreskin tissue embedded in OCT was cut into 5 µm sections using a cryostat and acetone fixed. Sections were stained with anti-IgA-biotin (Southern Biotech), and anti-CD138-PE (Miltenyi). Sections were washed, stained with streptavidin-FITC (eBioscience) and rewashed. Sections were then stained with DAPI to identify nuclei, mounted, and visualized on a Leica upright fluorescence microscope. Images were acquired with Openlab software. In order to calculate the density of specific cell subsets, the surface area of foreskin tissue sections was quantified using Volocity 3D Image Analysis Software software (PerkinElmer, Waltham MA).

### Study format and statistical analyses

The study format was a nested case-control study. Cases were defined as men who acquired HIV during the trial, and persistently HIV-seronegative controls were randomly selected from HIV-uninfected trial participants. Characteristics of cases and controls were compared at enrollment using Pearson Chi-square or Fisher's exact test. HIV IgA neutralization assay results were dichotomized into “neutralizing” and “non-neutralizing” based on an assay cutoff of ≥67% neutralizing capacity, as previously described [29], [53], [54]. To ensure that the results were robust, a sensitivity analysis using a neutralization capacity cutoff of ≥90% was also performed.

Univariate and multivariate logistic regression was used to assess the associations between IgA neutralization and the odds ratios (ORs) and 95% confidence intervals (95% CI) of HIV infection using the controls as the referent category. The ORs were estimated for HIV neutralization at enrollment to assess whether pre-existing neutralization affected subsequent risk of HIV acquisition. In addition, to assess IgA neutralization most proximate to the timing of HIV infection in cases, IgA neutralization at the last case HIV seronegative visit prior to detection of HIV infection, was assessed using the comparable study visit samples for the HIV-negative controls. These latter data were available for 93 cases and because controls could contribute more than one study visit, there were 164 control samples available. Within individual changes in IgA neutralization capacity over time were assessed to determine whether a change in neutralization capacity was associated with HIV infection. Covariates adjusted in the multivariable regression models were those sociodemographic and behavioral factors found to differ between cases and controls at enrollment (at P<0.10) and factors found to be associated with HIV risk in prior Rakai studies. The latter included age, education, marital status, and occupation, and time-dependent risk behaviors in the past year (number of sex partners, non-marital sex partners, condom use, alcohol use with sex), intercourse in the past week, and self-reported symptoms of GUD, discharge and dysuria. Robust variance estimates based on generalized estimating equations (GEE) were used to account for correlated data with repeated observations within individual participants. The level of AMPs in cases and controls were also assessed at enrollment and at the last case seronegative visit using the median and interquartile range (IQR) and mean (standard errors) of log_n_ transformed concentrations (log_n_ pg/mL). Samples with AMP levels below the level of detection were set to 1 pg/mL for these estimates. The odds ratios of HIV seroconversion were estimated for detectable versus undetectable levels of AMPs using logistic regression as described above. Statistical analyses used SAS 9.3, and statistical tests were two-sided with significance level of 0.05.

### Ethics statement

The study protocol was reviewed and approved by research ethics boards at the University of Toronto, the Uganda Virus Research Institute's Scientific and Ethical Committee, Karolinska Institutet and Western IRB (Olympia, WA). The clinical trial in which this case control study was nested was registered with ClinicalTrials.gov (NCT00425984). All subjects provided informed, written consent.
