# Correction: HIV Acquisition Is Associated with Increased Antimicrobial Peptides and Reduced HIV Neutralizing IgA in the Foreskin Prepuce of Uncircumcised Men

**DOI:** 10.1371/journal.ppat.1004515

**Published:** 2014-10-27

**Authors:** The PLOS Pathogens Staff

The affiliations for the ninth and fifteenth authors are incorrect. Maria J. Wawer and Ronald H. Gray are not affiliated with #1 but with #2 Department of Epidemiology, Johns Hopkins University, Bloomberg School of Public Health, Baltimore, Maryland, United States of America.
